# Evaluation of Methylation Status in the 5'UTR Promoter Region of the *DBC2* Gene as a Biomarker in Sporadic Breast Cancer

**Published:** 2012-06-13

**Authors:** Mehri Hajikhan Mirzaei, Mehrdad Noruzinia, Hamid Karbassian, Yousef Shafeghati, Mousa Keyhanee, Ali Bidmeshki-Pour

**Affiliations:** 1. Department of Cell Research Center and Medical Genetics, Sarem Women`s Hospital, Tehran, Iran; 2. Department of Biology, Science Faculty, Razi University, Kermanshah, Iran; 3. Department of Medical Genetics, Faculty of Medical Sciences, Tarbiat Modares University, Tehran, Iran; 4. Department of Surgery, Atiyeh Hospital, Tehran, Iran; 5. GRC, University of Social Welfare Sciences and Rehabilitation, Tehran, Iran

**Keywords:** Breast Neoplasm, DNA Methylation, *DBC2*, Iran

## Abstract

**Objective:**

Breast cancer is one of the most common malignancies in women worldwide. It is caused by a number of genetic and epigenetic factors. Aberrant hypermethylation of the promoter regions in specific genes is a key event in the formation and progression of breast cancers as well as the *DBC2* gene, as a tumor suppressor gene. Different studies show that the *DBC2* gene is inactivated through epigenetic mechanisms such as methylation in its promoter region. In this study, authors have tried to analyze methylation status in the promoter region of *DBC2* gene in affected women and healthy controls.

**Materials and Methods:**

In this experimental study, we evaluated the methylation status of *DBC2* gene with nested methylation-specific PCR (MSPCR) using specific methylated and unmethylated primer sets, in three separate PCR reactions. We used 50 tissue and blood samples of patients with breast cancer, 5 normal tissues and also 30 normal blood samples. Results were evaluated by the Mann-Whitney test, SPSS 16.0 statistical software.

**Results:**

Nested MSPCR results demonstrated that the frequency of the *DBC2* promoter region methylation status in tumor and blood samples of the affected patients was significantly higher than that of the corresponding normal controls.

**Conclusion:**

*DBC2* gene inactivation by methylation of CpG islands in the promoter region probably is a crucial step in the process of cell proliferation and susceptibility to different cancers, including breast cancer. Our study provides new evidence that aberrant methylation of the *DBC2* gene is involved in the tumorigenesis of breast cancer. DNA methylation in this gene is proven to be a potential marker for tumor diagnosis and prognosis, as well as a novel therapeutic target.

## Introduction

Breast cancer is one of the most common causes of morbidity and mortality in the world, accounting for 23% (1.38 million) of the total new cancer cases and 14% (458,400) of the total cancer deaths in 2008 (1). Two common genes (*BrCa1 and BrCa2*), have been identified in the hereditary breast cancer syndromes. However, hereditary susceptibility appears to account for only a minority of cases (2, 3). In general, breast cancer is thought to be a complex, multifactor disease resulting from the interaction of multiple genetic, epigenetic, and environmental factors (4).

Due to the high incidence of breast tumors and a lack of molecular markers for early diagnosis and prognosis, extensive efforts are needed to identify specific molecular markers for differentiating between non-tumoral and tumoral glands (5).

 One of these biomarker candidates, is the *DBC2* gene, which has been mapped and cloned on human chromosome 8 (8p21-22). *DBC2* inactivation, also known as *RHOBTB2*, is a tumor suppressor gene involved in breast cancer development (6). *DBC2* suppresses breast cancer cells proliferation through down-regulation of cyclin D1 (CCND1) (6). Further evaluation of the *DBC2* gene has suggested that it participates in different cellular pathways related to ubiquitination, cell cycle control, protein transport, apoptosis, and cyto-skeleton regulation (7-10).

In brief, biological studies have revealed that silencing and loss of function of the *DBC2* gene may precipitate breast cancer, and the reactivation of *DBC2* gene can lead to growth arrest of cancer cells (7). Several investigations have documented that the *DBC2* gene is frequently deleted in breast cancer, and the expression of this gene is silenced in breast and lung cancers (7). Moreover the silencing of this gene at the mRNA level is involved in the genesis and progression of breast cancer (9). Loss of heterozygosity (LOH), is the loss of normal function of one allele of a gene in which the other allele was already inactivated. LOH is the most common type of somatic alteration found in human breast tumors (11, 12). Numerous allelic analyses of variable polymorphic markers in breast cancer samples have demonstrated different LOH in different chromosomes including chromosome 8p (12-14).

Epigenetic changes (non-sequence-based alterations) are increasingly being considered as alternatives to mutations and chromosomal abnormalities (15). These include alterations in gene function through global DNA hypermethylation, histone modification and miRNAs. Most of the CpG dinucleotides are methylated on cytosine residues in vertebrate genomes, so it is necessary to test the tumor suppressor genes methylation status in various tumors in order to better understand their roles in carcinogenesis (16,17). Currently, researchers are investigating other genes that are methylated in different tumors, which might help in the early diagnosis of cancers.

In this study, we attempted to detect the methylation status of the *DBC2* gene promoter region in peripheral blood and breast tumor tissue samples of both affected and unaffected individuals by nested methylation-specific PCR (MSPCR).

## Materials and Methods

### Tissue and blood sampling

This study was approved with the Board of Ethics of Atiyeh Hospital, Tehran, Iran. All patients who participated in this study provided informed consent. Tumor samples were obtained from 50 patients who underwent surgery for breast cancer at Atiyeh Hospital, Tehran, Iran. The average age of the patients was 55 years, 7 months (range: 43-71 years). Patients were diagnosed with stages II and III breast cancer. All enrolled cases were sporadic; no other family members had been diagnosed with breast cancer. None of the patients received chemotherapy before surgery, and all tissues were pathologically confirmed as breast carcinoma. For control subjects, 5 normal breast tissue samples (average age: 41 years) and 30 normal blood samples (average age: 38 years) were used. Tissue samples preserved at -80 ºC. No phenotypic property was seen in the control samples.

### DNA preparation and bisulfite modification

The DNA from blood samples was extracted by the standard salting-out method (18) and DNA from breast tissue samples was extracted using animal tissue-based genomic DNA High Pure PCR Template Preparation Kit (Roche).

### Bisulfate modification

Treatment by bisulfite converts all unmethylated cytosine to uracil, which is recognized as thymidine by Taq polymerase; this process does not affect methylated cytosines. For this step, 10 µl of DNA (the typical A260/A280 ratio for isolated DNA samples with the Roche kit is 1.7-1.9) from each sample was used. We analyzed the presentation of DNA by electrophoresis after extraction. According to the protocol described with Tremblay KD, batches of DNA in 40 µl of water were denatured with 5.5 µl of 2 M sodium hydroxide for 10 minutes at 37℃. Thirty µl of 10 mM hydroquinone and 520 µl of 3 M sodium bisulfite were subsequently added. The DNA was overlaid with several drops of mineral oil, and the sample incubated at 50℃ for 16 hours. Afterwards, the procedure was followed according to the DNA High Pure PCR Template Preparation Kit (Roche) instructions.

DNA batches were de-sulfonated with 16.5 µl of 3 M sodium hydroxide for 10 minutes at room temperature and then neutralized with 51 µl of 10 M ammonium acetate. The DNA batches were then precipitated with 700 µl of absolute ethanol and 3 µl glycogen at -20℃ for 2 hours, washed twice with 70% ethanol, air-dried, and then re-suspended in 30 µl distilled water. Samples were stored at -20℃ until use.

### Nested methylation-specific PCR (MSPCR) analysis

For nested MSPCR, three sets of primers in three successive rounds were used. In the first round by using external primers, a 439 bp sequence was produced, which was larger than the target piece and contained the promoter region of the *DBC2* gene. In the second and third rounds, the other two sets of primers were used as methylated and unmethylated internal primers, wherein the methylated primers detected methylated regions and unmethylated primers were used to detect unmethylated regions. In these stages, two pieces of methylated and unmethylated 144 bp sequences, were produced. This section of the promoter contains numerous CpG islands.

The nested primer was designed with Methyl Primer Express software V1.0 (ABI, Foster City, CA) using GenBank AF315385 (19). The primer sequences are listed in table 1.

For the first round, 5 µl of bisulfite-treated DNA was added to bring the reaction volume up to 25 µl, and other reaction components are as follows: 1.5 µl dNTP (2 mM), 2 µl MgCl_2_, 0.75 µl Taq genomic DNA polymerase (CinnaGen) and 1 µl of the forward and reverse external primers.

**Table 1:** Sequences of external and internal primers, specific for methylated and unmethylated DNA of the promoter region of the DBC2 gene


Product size	Oligonucleotide primer sequences	Primer

439 bp	5' GGTGGTTTATTTGGTGATATTG 3'	DBC2 external (F)
	5' CCTACAACCTTACCTCC TAACAC 3'	DBC2 external (R)
		external
144 bp	5' GCGAGTTGGTATGTTATGTC 3'	DBC2 internal (F)
	5' TAATCTTACCCACGACGTTA 3'	DBC2 internal (R)
		methylated
144 bp	5' GGTGAGTTGGTATGTTATGTT 3'	DBC2 internal (F)
	5' CTAATCTTACCCAC AACATTA 3'	DBC2 internal (R)
		unmethylated


The PCR conditions were: 94℃ for 3 minutes followed by 35 cycles of 94℃ for 30 seconds, 60℃ for 1 minute, 72℃ for 50 seconds, and a final extension step of 72℃ for 10 minutes.

We used 1 µL of the PCR product for a second round of amplification, with methylated and unmethylated primers, and the following cycle parameters: 94℃ for 3 minutes, then 40 cycles of 94℃ for 30 seconds, 58℃ for 1 minute, (57℃ for the un-methylated primer), 72℃ for 50 seconds, followed by a final extension at 72℃ for 10 minutes.

PCR products were identified on 2% agarose gel electrophoresis. Amplifications with methylated primers that yielded the expected bands were considered methylation positive, and amplifications with both methylated and unmethylated primers that produced two bands were considered methylation negative. H_2_O was used as the negative control.

### Statistical analysis

Fifty patients with breast cancer were examined for *DBC2* gene methylation status by Nested MSPCR. The *DBC2* methylation status in tumors and normal tissues, along with study results on blood samples of the patients and controls, were analysed by the Mann-Whitney test, SPSS 16 statistical software. The obtained results demonstrated significant differences between affected individuals and normal controls in both types of samples.

**Table 2:** The results of nested MSPCR for affected and normal individuals in both tissues and blood.


	Methylated	Unmethylated	Methylated/ Unmethylated	p-value

Tumor tissue	17 (34%)	0	33 (66%)	0.467
Normal tissue	1 (20%)	0	4 (80%)	
Patients' blood samples	23 (46%)	0	27 (54%)	0.007
Normal blood samples	5 (16.6%)	3 (10%)	22 (73%)	


Amplifications with methylated primers that yielded expected bands were considered methylation positive; amplifications with both methylated and unmethylated primers and unmethylated that yielded two bands were considered methylation negative. Amplifications with unmethylated primers that yielded the expected bands were also considered methylation negative.

## Results

50 patients with breast cancer and normal individuals were examined for *DBC2* methylation by the nested MSPCR method. Patients' clinicopathological features were obtained from medical records, which included pathology reports.

**Fig 1:**
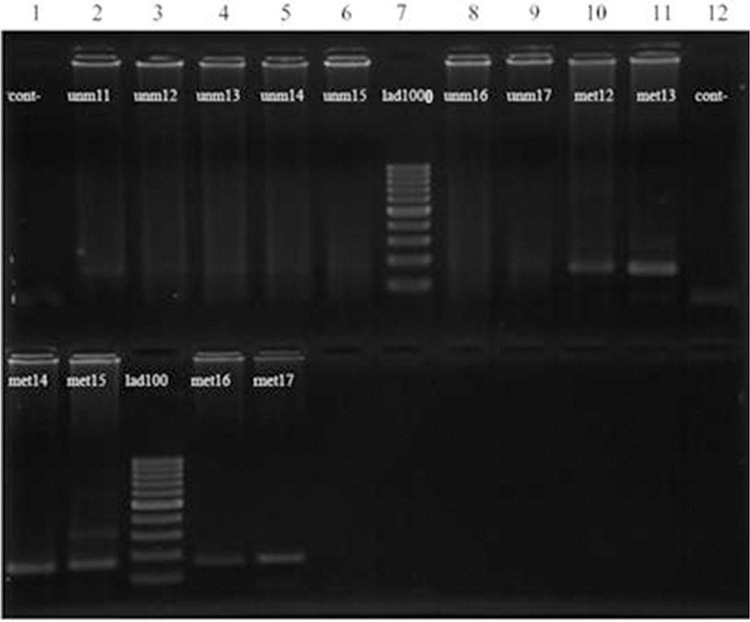
Results of nested MSPCR evaluation of DBC2 gene in blood samples in patients with breast cancer. Columns 2-9 (upper portion) represent the result of nested MSPCR of affected patients using unmethylated primers that did not show any bands. Columns 10 and 11 in the upper portion along with columns 1-5 in the lower portion belong to the same affected cases, using methylated primers. Column 7 is a 1000 bp ladder marker, column 1 is the negative control for unmethylated primers and column 12 is the negative control for methylated primers. We detected 23 methylation-specific bands in the blood samples of 50 affected women and 5 methylation-specific bands in the 30 normal control samples (p=0.007).

**Fig 2:**
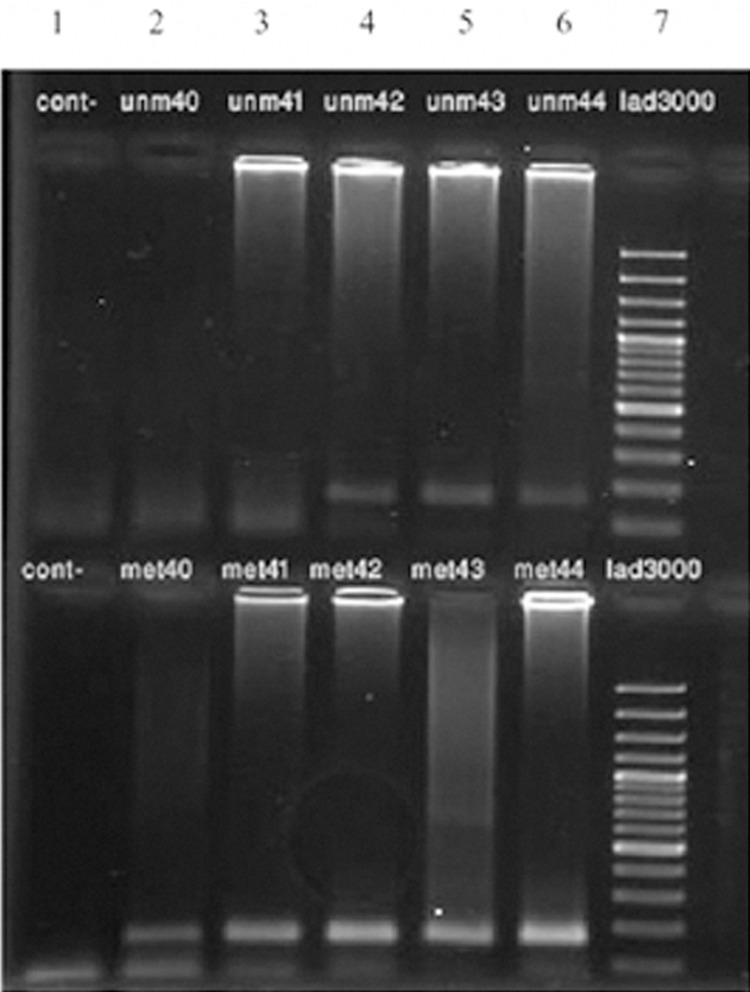
Results of nested MSPCR analyses of the DBC2 gene in tumor tissue samples in affected patients. In column 1 (upper and lower portion) distilled water was used as a negative control. Columns 2-6 show the results of samples 40-44. The upper row belongs to the unmethylated primers and lower row to the methylated primers. In the 50 affected tissue samples, 17 had only methylated bands and the remainder (33 samples) had both methylated and un-methylated bands.

We detected 23 methylation-specific bands in the whole blood samples of the 50 affected women and 5 methylation-specific bands in 30 normal controls (p=0.007, Fig 1). Of the 50 patient’s tissue samples, 17 had only methylated bands, while 33 had both methylated and unmethylated bands (Fig 2). In the 5 normal-tissue samples, one had only methylated bands, while the other 4 had both methylated and unmethylated bands (p=0.467). Our study demonstrated that the frequency of the hypermethylation of the *DBC2* promoter region in tumor and blood samples of affected individuals was significantly higher than that of corresponding normal controls (Table 2).

## Discussion

According to the World Health Organization statistics, worldwide, there are 7.6 million deaths annually due to cancer, of which around 500,000 are caused by breast cancer (20).

Most breast cancer cases are considered to be sporadic, and only 5% to 10% of the cases are estimated to be due to inherited susceptibility (21). Most of the sporadic cases, in turn, originate in a number of genetic and epigenetic aberrations (4). DNA methylation (the covalent addition of a methyl group to the cytosine base in the DNA) is an epigenetic event that affects cell function by altering gene expression. In principle, DNA methylation can silence gene expression by interfering with the sequence binding site of transcription factors or by producing more general effects on the chromatin (6, 7, 12).

With the increasing number of genes known to be silenced as a result of hypermethylation of their promoters, attempts have been made to identify the sets of genes that are methylated only in particular tumors (22). For those genes that were found to be down regulated in breast cancer, it is possible that DNA methylation is one mechanism that plays an important role in their silencing (23).

There are several techniques for detecting methylation patterns. MSPCR is a fast, inexpensive, and accurate technique used for methylation status studies. This technique relies on the alteration that sodium bisulfite induces in DNA sequences and the difference it creates between methylated and unmethylated cytosine by the deamination of unmethylated cytosine to uracil. Then the methylation pattern can be detected by using the specific methylated and unmethylated primer sets in two separate PCR reactions.

These epigenetic alterations can produce early loss of cell cycle control, deregulation of gene transcription factors, disrupt cell-to-cell interactions, and induce multiple types of genetic instability, which are all characteristic of neoplasia (24). Numerous genes have been found to undergo hypermethylation in cancer, including genes involved in cell cycle regulation, DNA repair, apoptosis, drug resistance, detoxification, differentiation, angiogenesis, and metastasis (25). Biological studies have revealed that *DBC2* gene reactivation can lead to the growth arrest of breast tumor tissue. Further analysis of the *DBC2* gene has suggested its participation in cellular pathways related to ubiquitination, cell cycle control, protein transport, apoptosis, and cytoskeleton regulation (9, 26). *DBC2* gene inactivation is thus a crucial step in the process of cell proliferation, and its reactivation can lead to the growth arrest of cancer cells (7). Other studies on *DBC2* expression imply that this gene becomes inactivated during breast carcinoma (10).

## Conclusion

Our study provides evidence for the first time that the aberrant methylation of the *DBC2* gene is involved in at least the tumorigenesis of sporadic breast cancer. Since DNA methylation is known to be a potential marker for early tumor diagnosis and prognosis, our findings will be useful in the early detection and epigenetic therapy of breast cancer (15). Additional investigations are required to elucidate the feasibility of *DBC2* gene methylation analysis for clinical application.

## References

[1] Jemal A, Bray F, Center MM, Ferlay J, Ward E, Forman D (2011). Global cancer statistics. CA Cancer J Clin.

[2] Couch FJ, Weber BL, Vogelstein B, Kinzler KW (1998). Breast cancer. The genetic basis of human cancer.

[3] Ellisen LW, Haber DA (1998). Hereditary breast cancer. Annu Rev Med.

[4] Nathanson KL, Wooster R, Weber BL (2001). Breast cancer genetics: what we know and what we need. Nat Med.

[5] Shiry S, Hoseinpourfeizi MA, Babaei E, Ghanbarian M (2011). Evaluating expression pattern of KLF4 as molecular biomarker in breast cancer. Cell jouanal (Yakhteh).

[6] Yoshihara T, Collado D, Hamaguchi M (2007). Cyclin D1 down-regulation is essential for DBC2's tumor suppressor function. Biochem Biophys Res Commun.

[7] Hamaguchi M, Meth JL, Von Klitzing C, Wei W, Esposito D, Rodgers L (2002). DBC2, a candidate for a tumor suppressor gene involved in breast cancer. Proc Natl Acad Sci USA.

[8] Siripurapu V, Meth J, Kobayashi N, Hamaguchi M (2005). DBC2 significantly influences cell-cycle, apoptosis, cytoskeleton and membrane- trafficking pathways. J Mol Biol.

[9] Chang FK, Sato N, Kobayashi-Simorowski N, Yoshihara T, Meth JL, Hamaguchi M (2006). DBC2 is essential for transporting vesicular stomatitis virus glycoprotein. J Mol Biol.

[10] Bi Y, Wei L, Mao HT, Zhang L, Zuo WS (2008). Expressions of Fas, CTLA-4 and RhoBTB2 genes in breast carcinoma and their relationship with clinicopathological factors. Zhonghua Zhong Liu Za Zhi.

[11] Miller BJ, Wang D, Krahe R, Wright FA (2003). Pooled analysis of loss of heterozygosity in breast cancer: a genome scan provides comparative evidence for multiple tumor suppressors and identifies novel candidate regions. Am J Hum Genet.

[12] Callahan R, Cropp C, Sheng ZM, Merlo G, Steeg P, Liscia D (1993). Definition of regions of the human genome affected by loss of heterozygosity in primary human breast tumors. J Cell Biochem Suppl.

[13] Callahan R (1998). Somatic mutations that contribute to breast cancer. Biochem Soc Symp.

[14] Lasko D, Cavenee W, Nordenskjold M (1991). Loss of constitutional heterozygosity in human cancer. Annu Rev Genet.

[15] Feinberg AP, Ohlsson R, Henikoff S (2006). The epigenetic progenitor origin of human cancer. Nat Rev Genet.

[16] Lu TY, Kao CF, Lin CT, Huang DY, Chiu CY, Huang YS (2009). DNA methylation and histone modification regulate silencing of OPG during tumor progression. J Cell Biochem.

[17] Chuang JC, Jones PA (2007). Epigenetics and MicroRNAs. Pediatr Res.

[18] Miller SA, Dykes DD, Polesky HF (1988). A simple salting out procedure for extracting DNA from human nucleated cells. Nucleic Acids Res.

[19] Shi Y, Chen JY, Yang J, Li B, Chen ZH, Xiao CG (2008). DBC2 gene is silenced by promoter methylation in bladder cancer. Urol Oncol.

[20] Jelen L, Fevens T, Krzyzak A (2008). Classification of breast cancer malignancy using cytological images of fine needle aspiration biopsies. Int J Appl Math Comput Sci.

[21] Bissonauth V, Shatenstein B, Ghadirian P (2008). Nutrition and breast cancer among sporadic cases and gene mutation carriers: an overview. Cancer Detect Prev.

[22] Zemliakova VV, Zhevlova AI, Strelnikov VV, Liubchenko LN, Vishnevskaia IaV, Tretiakova VA (2003). Abnormal methylation of several tumor suppressor genes in sporadic breast Cancer. Mol Biol(Mosk).

[23] Balfe PJ, McCann AH, Welch HM, Kerin MJ (2004). Estrogen receptor beta and breast cancer. Eur J Surg Oncol.

[24] Hiltunen MO, Alhonen L, Koistinaho J, Myohanen S, Paakkonen M, Marin S (1997). Hypermethylation of the APC (adenomatous polyposis coli) gene promoter region in human colorectal carcinoma. Int J Cancer.

[25] Issa JP (2004). CpG island methylator phenotype in cancer. Nat Rev Cancer.

[26] Wilkins A, Ping Q, Carpenter CL (2004). RhoBTB2 is a substrate of the mammalian Cul3 ubiquitin ligase complex. Genes Dev.

